# The first reported case of a patient with pancreatic cancer treated with cone beam computed tomography-guided stereotactic adaptive radiotherapy (CT-STAR)

**DOI:** 10.1186/s13014-022-02125-z

**Published:** 2022-09-13

**Authors:** Minsol Kim, Joshua P. Schiff, Alex Price, Eric Laugeman, Pamela P. Samson, Hyun Kim, Shahed N. Badiyan, Lauren E. Henke

**Affiliations:** 1 Department of Electrical and Computer Engineering, School of Engineering and Applied Science, University of Virginia, 351 McCormick Rd, Charlottsville, VA 22904 USA; 2 Department of Radiation Oncology, Washington University School of Medicine in St. Louis, 4921 Parkview Place, Campus Box 8224, St. Louis, MO 63110 USA

**Keywords:** Pancreatic cancer, SBRT, Image guided radiation therapy, CT

## Abstract

**Background:**

Online adaptive stereotactic radiotherapy allows for improved target and organ at risk (OAR) delineation and inter-fraction motion management via daily adaptive planning. The use of adaptive SBRT for the treatment of pancreatic cancer (performed until now using only MRI or CT on rails-guided adaptive radiotherapy), has yielded promising outcomes. Herein we describe the first reported case of cone beam CT-guided stereotactic adaptive radiotherapy (CT-STAR) for the treatment of pancreatic cancer.

**Case presentation:**

A 61-year-old female with metastatic pancreatic cancer presented for durable palliation of a symptomatic primary pancreatic mass. She was prescribed 35 Gy/5 fractions utilizing CT-STAR. The patient was simulated utilizing an end-exhale CT with intravenous and oral bowel contrast. Both initial as well as daily adapted plans were created adhering to a strict isotoxicity approach in which coverage was sacrificed to meet critical luminal gastrointestinal OAR hard constraints. Kilovoltage cone beam CTs were acquired on each day of treatment and the radiation oncologist edited OAR contours to reflect the patient’s anatomy-of-the-day. The initial and adapted plan were compared using dose volume histogram objectives, and the superior plan was delivered. Use of the initial treatment plan would have resulted in nine critical OAR hard constraint violations. The adapted plans achieved hard constraints in all five fractions for all four critical luminal gastrointestinal structures.

**Conclusions:**

We report the successful treatment of a patient with pancreatic cancer treated with CT-STAR. Prior to this treatment, the delivery of ablative adaptive radiotherapy for pancreatic cancer was limited to clinics with MR-guided and CT-on-rails adaptive SBRT technology and workflows. CT-STAR is a promising modality with which to deliver stereotactic adaptive radiotherapy for pancreatic cancer.

**Supplementary Information:**

The online version contains supplementary material available at 10.1186/s13014-022-02125-z.

## Background

Pancreatic cancer is a lethal malignancy with a five-year overall survival rate of 2–10% [1–4]. In recent years, there has been an increased focus on the utilization of stereotactic body radiotherapy (SBRT) for the definitive treatment of pancreatic malignancies [2, 5, 6]. SBRT for pancreatic cancer is also critical in the palliative setting, as SBRT has been demonstrated to elicit durable local control and long-lasting relief of symptoms of local progression such as abdominal pain and gastric outlet obstruction [7–9]. However, the delivery of SBRT for pancreatic tumors is challenging given the close proximity of the mobile and radiosensitive luminal gastrointestinal tract [10]. Magnetic resonance imaging (MRI) guided radiotherapy has been shown to allow precise delineation of daily target and organ at risk (OAR) volumes, improving the efficacy of pancreatic SBRT while minimizing toxicity [11–13]. Recently, the implementation of daily online adaptive planning via stereotactic magnetic resonance guided adaptive radiotherapy (SMART) has yielded promising progression-free and overall survival rates as well as a favorable toxicity profile in the ablation of pancreatic cancer [4,14–16].

Recently, a novel ring gantry computed tomography (CT) based radiotherapy machine has been developed with a high-quality cone-beam CT capable of yielding high resolution on-board volumetric images and an artificial intelligence (AI) enhanced treatment planning system (TPS), which is capable of daily adaptive planning (ETHOS, Varian Medical Systems, Palo Alto, CA) [17–19]. The use of cone beam CT-guided adaptive radiotherapy for the clinical ablation of pancreatic cancer has not yet been described. Herein we describe the first reported treatment of a patient with pancreatic cancer using cone beam CT-guided stereotactic adaptive radiotherapy (CT-STAR), including a discussion of the workflow and dosimetric analysis of the treatment.

## Case presentation

### Patient presentation

**Fig. 1:**
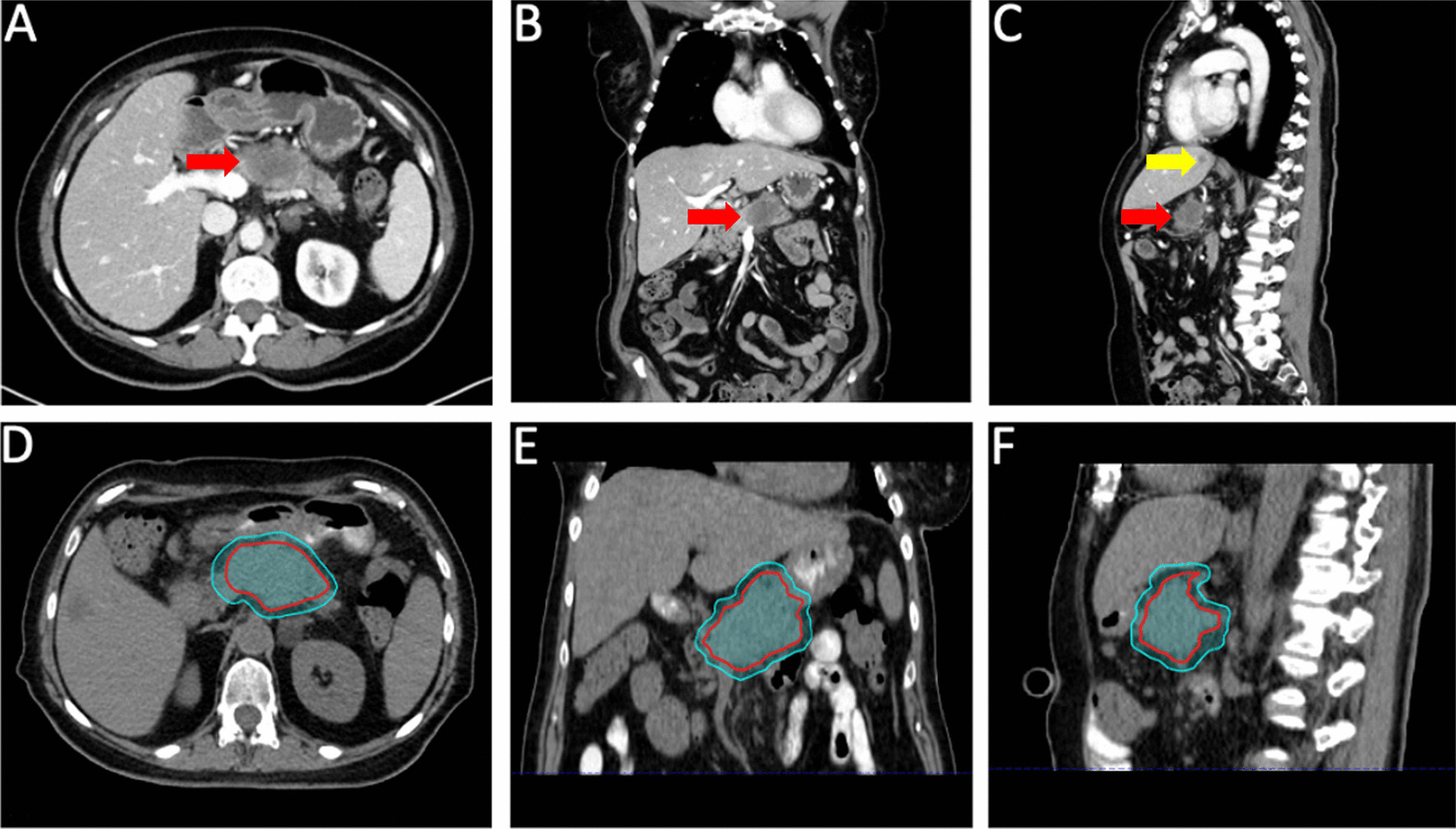
Pancreatic tumor at time of presentation to radiation oncology. Axial, coronal, and sagittal diagnostic (**A**–**C**) as well as simulation (**D**–**F**) CT images of the patient at time of presentation to radiation oncology. The primary tumor is indicated on the diagnostic images by the red arrow and a liver metastasis is indacted by the yellow arrow. The GTV (red contour) and PTV (cyan contour) are delineated on the CT simulation images

A 61-year-old woman presented following an episode of abdominal pain due to acute pancreatitis. During the patient’s work up, a CT chest/abdomen/pelvis demonstrated a mass in the pancreatic body. Biopsy of the mass confirmed pancreatic adenocarcinoma. The patient met with medical oncology and was recommended neo-adjuvant systemic therapy but declined and pursued alternative therapies. The patient returned to clinic several months later with abdominal pain and interval imaging demonstrating progression of local disease with encasement of the splenic and superior mesenteric veins (Fig. 1) as well as the development of liver metastases. The primary mass measured 4.8 × 3.8 cm. The patient was referred to radiation oncology for consideration of palliative radiotherapy. On interview, the patient reported left upper quadrant and back pain as well as malaise and weight loss. Physical exam was otherwise unremarkable. The patient was recommended SBRT to her primary mass for durable palliation, 35 Gy in 5 fractions, 7 Gy per fraction. Given the high dose per fraction and adjacent critical organs at risk, the treating radiation oncologist elected to use daily online adaptation with cone beam CT-guidance.

### Treatment planning and delivery

The patient was simulated utilizing an end-exhale breath-hold CT with intravenous and oral bowel contrast and a 4-dimensional CT. Intravenous contrast was administered at the 45-s delay phase per institutional protocol. The primary image used for planning was the end-exhale breath-hold CT. The 4D-CT is captured in the case that the patient is non-compliant with breath-hold and requires treatment with a different modality and/or dose and fractionation. Of note, as contrast is not delivered with each subsequent daily cone beam CT, the density of the contrast is overrided on the simulation CT to the density of water so that the contrast has no dosimetric impact on the initial plan (P_I_). The patient was positioned in a custom immobilization device with left arm down and right arm up, per institutional pancreatic SBRT practice. An MRI was obtained at time of simulation and fused to the simulation images for assistance in target delineation. All treatment planning was performed in the ETHOS (v.02.01.00) TPS. The gross tumor volume (GTV) comprised the gross tumor demonstrated on simulation imaging. As the patient was simulated and intended to be treated at end-exhale breath-hold, a internal GTV or internal target volume was not created. No clinical target volume (CTV) was utilized per standard institutional pancreatic SBRT practice. A 0.5 cm uniform volumetric expansion was applied to form a planning target volume (PTV). The relevant organs-at-risk (OARs) were contoured at the axial slices from 3 cm below to 3 cm above the PTV.

A PTV optimization (PTV_opt_) structure was generated, made from the PTV minus any overlap with critical OARs plus a 5 mm margin on the OARs. The critical OARs were the luminal gastrointestinal structures, namely the stomach, duodenum, small bowel, and large bowel. This PTV_opt_ was used to drive prescription dose to the tumor to drive target coverage, given that areas of direct PTV and OAR overlap are not prioritized for target coverage per our standard adaptive radiotherapy practices [4, 20–22]. Both the P_I_ and adaptive plans (P_A_) were generated using a strict isotoxicity approach, in which maximum OAR constraints are prioritized over target coverage [21, 23]. However, a minimum dose of 25 Gy was maintained to the PTV to ensure some uncertainty margin coverage. Dose constraints and objectives are in Table 1. Conservative luminal gastrointestinal OAR constraints were used given the palliative nature of the case. We have provided our standard departmental pancreatic adaptive SBRT dose constraints in Additional file 1: Table S1. A beam arrangement of two ¾ co-planar arcs was used, with 30 and 330 degree collimator angles.

**Table 1:** OAR constraint and target volume metrics are presented for the initial non adaptive (P_I_) and adapted (P_A_) plans

Organ-at-risk	Strict constraint	P_I_ mean (std dev)	P_I_ median (range)	P_A_ mean (std dev)	P_A_ median (range)
Stomach	V25 Gy < 0.5 cc (cc)	10.2 (3.6)	9.7 (5.6–14.8)	0.0 (0.1)	0.0 (0.0–0.1)
Duodenum	V25 Gy < 0.5 cc (cc)	0.1 (0.1)	0.1 (0.0–0.2)	0.0 (0.0)	0.0 (0.0–0.0)
Small bowel	V25 Gy < 0.5 cc (cc)	3.8 (5.1)	1.2 (0.3–13.8)	0.0 (0.0)	0.0 (0.0–0.1)
Large bowel	V25 Gy < 0.5 cc (cc)	0.0 (0.0)	0.0 (0.0–0.1)	0.1 (0.1)	0.0 (0.0–0.2)
Liver	V25 Gy < 33% (%)	9.5 (1.6)	10.1 (6.8–11.4)	12.1 (1.8)	11.0 (10.3–14.7)
	700 cc < 20 Gy (Gy)	0.4 (.1)	0.3 (0.2–0.6)	0.4 (0.1)	0.4 (0.3–0.5)
	Mean < 20 Gy (Gy)	1.0 (0.2)	1.0 (0.8–1.2)	1.2 (0.1)	1.2 (1.0–1.3)
Spinal cord	V25 Gy < 0.5 cc (cc)	0.0 (0.0)	0.0 (0.0–0.0)	0.0 (0.0)	0.0 (0.0–0.0)
Kidneys (both)	Mean < 18 Gy (Gy)	0.8 (0.7)	0.8 (0.7–0.9)	0.8 (0.1)	0.8 (0.7–0.9)

Target volume	Coverage goal	P_I_ mean (std dev)	P_I_ median (range)	P_A_ mean (std dev)	P_A_ median (range)
PTV V100	N/A (%)	77.1 (2.1)	76.0 (74.5–80.0)	77.8 (5.1)	80.8 (67.9–81.3)
PTV D95	N/A (Gy)	4.7 (0.1)	4.6 (4.6–4.9)	4.8 (0.1)	4.8 (4.6–5.0)
PTV_opt_	95% (%)	83.5 (3.8)	83.5 (78.8–91.4)	99.6 (0.3)	99.5 (99.2–100.1)
GTV V100	N/A (%)	89.9 (1.1)	90.0 (88.5–91.4)	90.0 (4.0)	91.8 (82.2–93)
GTV D95	N/A (Gy)	6.0 (0.2)	6.1 (5.6–6.3)	6.4 (0.3)	6.5 (5.7–6.7)

Mean and median constraint and target metrics for the P_I_ represent the hypothetical use of the P_I_ applied to all five fractions

*N/A* not applicable, *Std Dev* standard deviation

Daily P_A_ were created based on the patient’s anatomy-of-the-day. The TPS automatically deformed the OAR and target contours from the P_A_ onto the daily cone beam CT using a vendor supplied elastic deformation algorithm, and the TPS AI auto-adjusted the stomach, duodenum, and liver according to the anatomy-of-the-day. The deformed GTV was then overwritten and the simulation GTV was ridigly copied onto the patient’s anatomy-of-the-day. OARs within a 3-cm contour ring (per standard adaptive protocol [22]) were adjusted by the radiation oncologist in order to confirm accuracy. The initial simulation based treatment plan (P_I_) was projected on the patient anatomy-of-the-day at the same time that the re-optimized daily adapted plan (P_A_) was generated. The P_I_ and P_A_ were compared using dose volume histogram (DVH) objectives, and the superior plan that met all dosimetric goals was delivered. Of note, all acquired kV cone beam CTs were considered of sufficient quality for target and OAR delineation as well as daily adaptation per the treating radiation oncologist and medical physicist.

### Dosimetric and clinical results

**Fig. 2:**
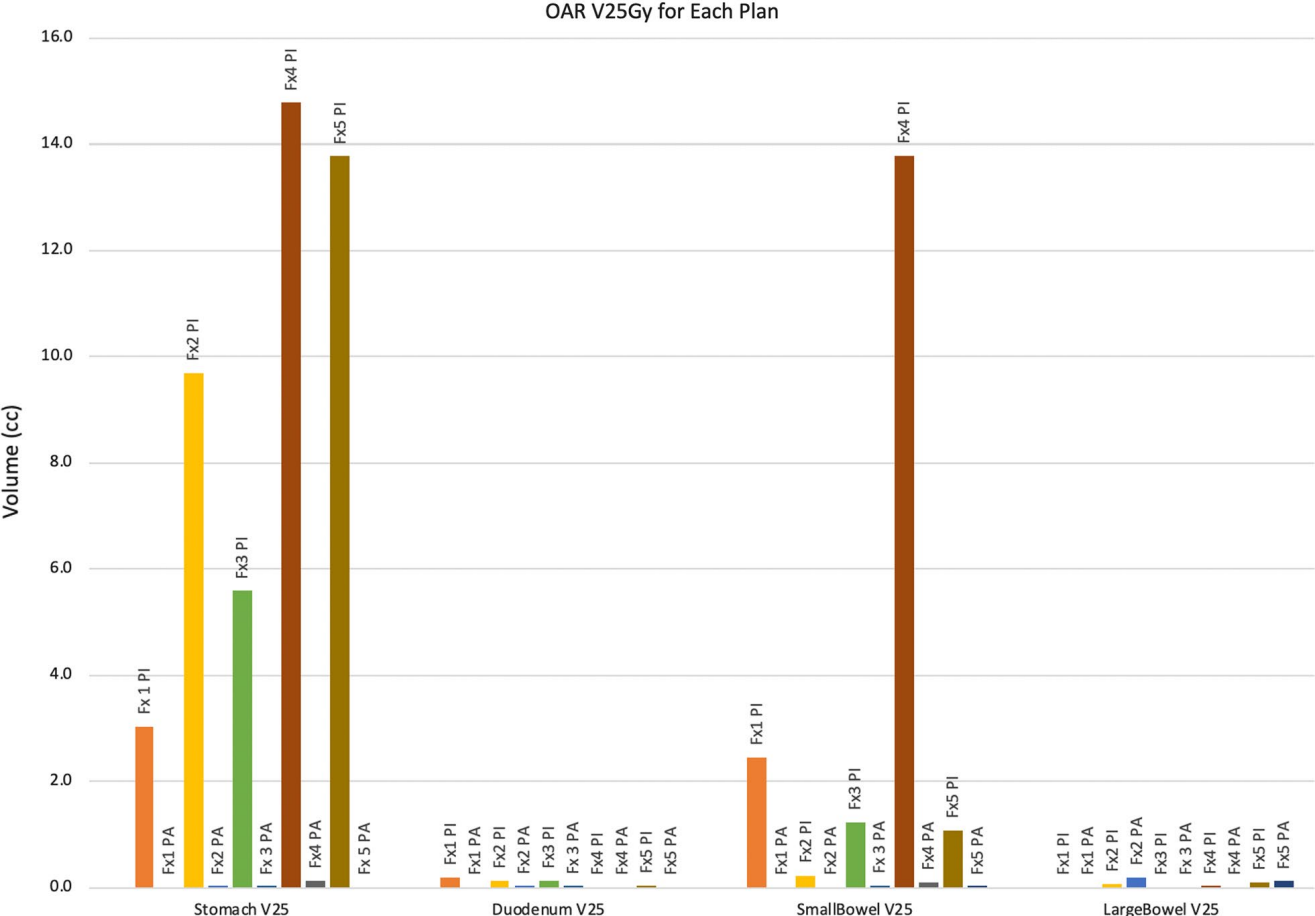
V25 (cc) of initial and adaptive plans of critical organs at risk. The V25 of the initial (P_I_) and adaptive (P_A_) plans for the critical luminal gastrointestinal OARs. Y-axis is in cc. Delivery of the initial plan would have yielded nine OAR hard constraint violations. Adaptive planning was able to meet hard constraints for all OARs in all five fractions. Fx = fraction

**Fig. 3:**
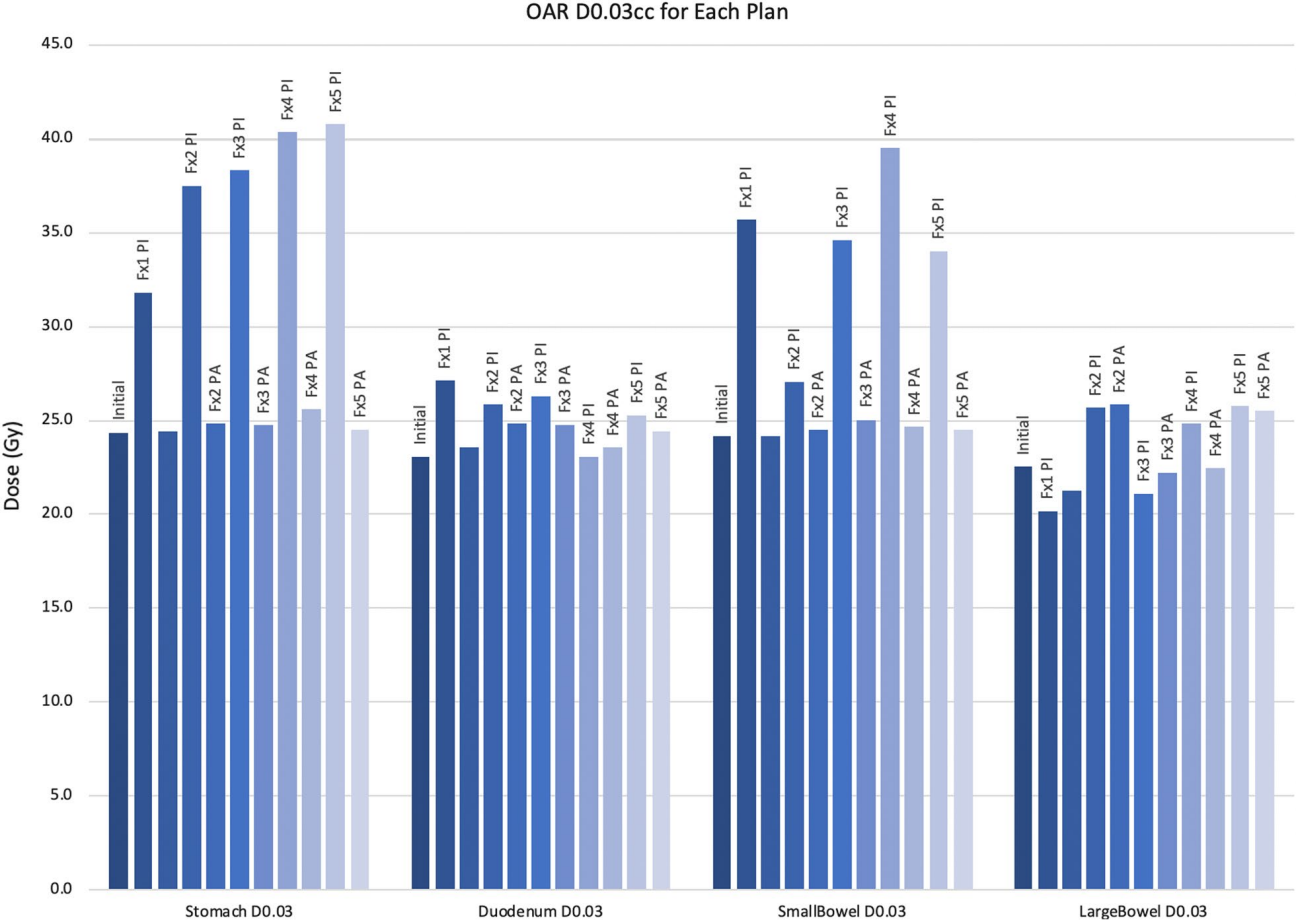
Maximum doses of critical OARs. The D_max_ values of the initial (P_I_) and adaptive (P_A_) plans for critical luminal gastrointestinal OARs. Y-axis is in Gy. Adaptive planning yielding substantial D_max_ reductions for the stomach and small bowel. Fx = fraction

**Fig. 4:**
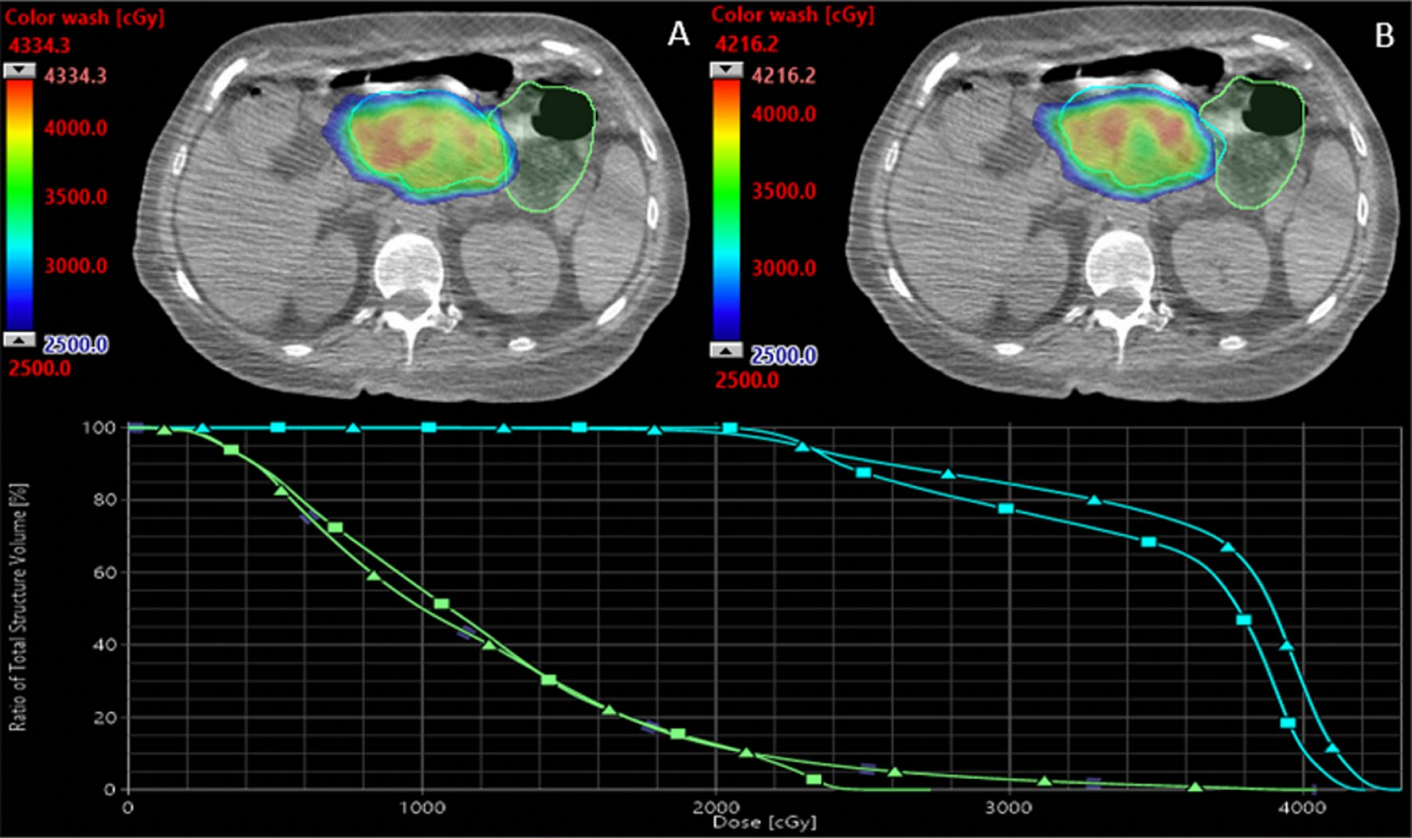
Initial and adaptive plan comparison. An initial (**A**) and adaptive plan (**B**) for a single fraction of radiotherapy. In the initial plan, the high dose color wash (> 25 Gy) is in the small bowel (light green), whereas in the adapted plan, the high dose color wash does not enter the small bowel. The DVH demonstrates the dose delivered to the small bowel as well as the PTV (cyan) in the initial (triangle) and adaptive plans (square)

Constraint and coverage metrics for the P_I_ and P_A_ are demonstrated in Table 1. Mean PTV and GTV D95 for all five fractions was 23.25 Gy and 30.20 Gy in the P_I_ and 24.11 Gy and 31.85 Gy in the P_A_, respectively. Dosimetric parameters, specifically the volume received 25 Gy (V25) and maximum dose (D_max_) for critical luminal gastrointestinal structures, are demonstrated in Figs. 2 and 3. The use of the P_I_ would have resulted in violation of the stomach hard constaint in all five fractions, and violation of the small bowel constraint in four of five fractions (Fig. 2). The P_A_ achieved hard constraints in all five fractions for all four critical luminal gastrointestinal structures. Figure 4 illustrates how the use of daily adaptive planning allowed for a specific radiotherapy fraction to achieve the small bowel hard constraint, where as delivery of the P_I_ would have violated that constraint.

Treatment component times were recorded and are demonstrated in Table 2. Mean (standard deviation) total treatment time was 70 min (68.3–81.7) and treatment time decreased each consecutive fraction. The patient completed all five fractions of CT-STAR without issue. The patient ultimately progressed locally and distantly, and passed away several months after treatment.

**Table 2:** Treatment component times are presented for each fraction

Treatment component	Fraction 1	Fraction 2	Fraction 3	Fraction 4	Fraction 5	Mean	Standard deviation
Patient setup	18	9	17	10	N/R	13.5	4.7
CBCT time	1	5	1	2	1	2	1.7
Contouring	39	28	17	19	27	26	8.7
Plan re-optimization	3	7	6	7	6	5.8	1.6
Plan review	< 1	< 1	< 1	1	1	0.4	0.5
Quality assurance	8	3	6	2	2	4.2	2.7
Pre-treatment CBCT	1	2	5	3	4	3	1.6
Beam delivery	26	21	8	9	14	15.6	7.8
Patient exit	1	4	1	7	5	3.6	2.6
Total	86	79	63	62	60	70	11.7

Times are in minutes

*N/R* not recorded

## Discussion and conclusions

### Discussion

Herein we describe the first reported use of CT-STAR for the treatment of a patient with pancreatic cancer using a novel ring gantry device. These data demonstrate that the delivery of the P_I_ would have led to nine critical OAR hard constraint violations across all five fractions, and that the daily P_A_ met all critical OAR hard constraints in all five fractions. Furthermore, the use of daily adaptation improved PTV_opt_, GTV V100, and GTV D95 coverage (Table 1) while alleviating the hard constraint violations. With regards to workflow, the overall treatment times were within the range of previously described treatment times for daily adaptation, and the decreased time per each consecutive fraction suggests that treatment times decrease with increased patient/staff familiarity [22, 24].

The utility of adaptive stereotactic radiotherapy for the treatment of pancreatic cancer can not be understated. The effective ablation of pancreatic cancers requires the delivery of biologic effective dose of at least 100 Gy [10]. However, this is difficult to achieve as the pancreas is adjacent to several mobile and radiosensitive OARs. Initial studies evaluating the use of ablative doses of standard CT-guided stereotactic radiotherapy for the treatment of pancreatic cancer proved efficacious with regards to local control, but also displayed high rates of luminal gastrointestinal organ toxicity [25–27]. Adaptive radiotherapy can improve the therapeutic index of SBRT for pancreatic cancer. Recently, our institution published outcomes for patients with inoperable pancreatic cancer treated with SMART and demonstrated durable progression-free and overall survival rates as well as a favorable toxicity profile [4]. While these data are promising, it’s notable that their application is limited to MR-guided workflows. Prior to the advent of the ETHOS platform, adaptive SBRT for pancreatic cancer was limited to clinics with MR-guided or CT-on-rails workflows [4, 16, 28, 29].

While CT-STAR has the capacity to expand access to adaptive pancreatic SBRT, there are potential limitations of using a CBCT-guided platform instead of a MR-guided platform. The improved soft tissue contrast of MRI can be useful in pancreatic and abdominal contouring, which can be of particular importance when delineating gross organ invasion. In contrast with MR-guidance, gross organ invasion is challenging to delineate on cone beam CT. In our experience [4], approximately 10% of patients with locally advanced pancreatic cancer present with evidence of gross organ, and the use of CBCT-guided adaptive SBRT may be limited in that subset of patients. This may be of consideration when planning to install either a MR- or cone beam CT-guided adaptive platform.

Herein we demonstrate that stereotactic adaptive radiotherapy is able to be delivered on a cone beam CT-guided modality, which promises to increase access to adaptive pancreatic SBRT world wide. This case presentation demonstrates the potential for CT-STAR to provide a additional avenue for radiation oncologists to ablate pancreatic cancer.

### Conclusions

CT-STAR is a viable modality for the delivery of adaptive stereotactic radiotherapy for the ablation of pancreatic cancer. Clinical trials are warranted to investigate the impact of this modality on overall and progression-free survival as well as toxicity.

## Supplementary Information

**Additional file 1: Table S1.** Case report and standard OAR constraints. The constraints used for the patient in this case report and our standard departmental pancreatic adaptive SBRT dose constraints are demonstrated. The standard luminal gastrointestinal OAR constraints are in bold.

## Data Availability

Research data are stored in an institutional repository and will be shared upon request to the corresponding author.

## Abbreviations

AI: Artificial intelligence
CBCT: Cone beam computed tomography.
CT: Computed tomography
CT-STAR: CT-guided stereotactic adaptive radiotherapy
CTV: Clinical target volume
D_max_: Maximum dose recieved
DVH: Dose volume histogram
GTV: Gross tumor volume
MRI: Magnetic resonance imaging
OAR: Organ at risk
P_A_: Adapted plan
P_I_: Initial plan
PTV: Planning target volume
PTV_opt_: PTV optimization structure
SBRT: Stereotactic body radiation therapy
SMART: Stereotactic magnetic resonance guided adaptive radiotherapy
TPS: Treatment planning system
V25: Volume receiving 25 Gy
