# Supplementary material for: The first reported case of a patient with pancreatic cancer treated with cone beam computed tomography-guided stereotactic adaptive radiotherapy (CT-STAR)
Source: Radiat Oncol. 2022 Sep 13;17:157. doi: 10.1186/s13014-022-02125-z (PMC9472353; doi:10.1186/s13014-022-02125-z)
Supplement: Supplementary file 1 — Additional file 1: Table S1. Case report and standard OAR constraints. The constraints used for the patient in this case report and our standard departmental pancreatic adaptive SBRT dose constraints are demonstrated. The standard luminal gastrointestinal OAR constraints are in bold. [file 13014_2022_2125_MOESM1_ESM.docx]

Supplementary File

| **Organ-at-Risk** | **Case Report Constraint** | **Standard Constraint** |
| --- | --- | --- |
| Stomach | V25 Gy < 0.5 cc (cc) | **V36Gy < 0.5cc (cc)** |
| Duodenum | V25 Gy < 0.5 cc (cc) | **V36Gy < 0.5cc (cc)** |
| Small bowel | V25 Gy < 0.5 cc (cc) | **V36Gy < 0.5cc (cc)** |
| Large bowel | V25 Gy < 0.5 cc (cc) | **V36Gy < 0.5cc (cc)** |
| Liver | V25 Gy < 33% (%) | V25 Gy < 33% (%) |
|  | 700 cc < 20 Gy (Gy) | 700 cc < 20 Gy (Gy) |
|  | Mean < 20 Gy (Gy) | Mean < 20 Gy (Gy) |
| Spinal cord | V25 Gy < 0.5 cc (cc) | V25 Gy < 0.5 cc (cc) |
| Kidneys (both) | Mean < 18 Gy (Gy) | Mean < 18 Gy (Gy) |

Supplementary Table 1. **Case report and standard OAR constraints.** The constraints used for the patient in this case report and our standard departmental pancreatic adaptive SBRT dose constraints are demonstrated. The standard luminal gastrointestinal OAR constraints are in **bold**.
